# Chemokine CCL4 Induces Vascular Endothelial Growth Factor C Expression and Lymphangiogenesis by miR-195-3p in Oral Squamous Cell Carcinoma

**DOI:** 10.3389/fimmu.2018.00412

**Published:** 2018-03-02

**Authors:** Ming-Yu Lien, Hsiao-Chi Tsai, An-Chen Chang, Ming-Hsui Tsai, Chun-Hung Hua, Shih-Wei Wang, Chih-Hsin Tang

**Affiliations:** ^1^Division of Hematology and Oncology, Department of Internal Medicine, China Medical University Hospital, Taichung, Taiwan; ^2^Graduate Institute of Basic Medical Science, China Medical University, Taichung, Taiwan; ^3^Department of Scientific Education, Qinghai Red Cross Hospital, Xining, Qinghai, China; ^4^Institute of Biomedical Sciences, National Chung Hsing University, Taichung, Taiwan; ^5^Department of Otolaryngology, China Medical University Hospital, Taichung, Taiwan; ^6^Department of Otorhinolaryngology, China Medical University Hospital, Taichung, Taiwan; ^7^Department of Medicine, Mackay Medical College, New Taipei City, Taiwan; ^8^College of Pharmacy, Graduate Institute of Natural Products, Kaohsiung Medical University, Kaohsiung, Taiwan; ^9^Department of Biotechnology, College of Health Science, Asia University, Taichung, Taiwan

**Keywords:** CCL4, miR-195-3p, vascular endothelial growth factor C, lymphangiogenesis, human oral squamous cell carcinoma

## Abstract

The inflammatory chemokine (C–C motif) ligand 4 (CCL4) plays an important role in the pathogenesis and progression of cancer. In particular, higher serum CCL4 levels in patients with oral squamous cell carcinoma (OSCC) are associated with a more advanced stage of disease. OSCC accounts for approximately 95% of oral cancer in Taiwan and has a poor prognosis, due to aggressive local invasion and metastasis, leading to recurrence. OSCC spreads preferentially through lymphatic vessels and has the propensity to metastasize to the cervical lymph nodes even in the early stage of disease. Vascular endothelial growth factor C (VEGF-C) is an essential regulator of lymphangiogenesis. In particular, VEGF-C is specific to lymphatic vessel development, and VEGF-C expression levels have been found to directly correlate with lymph node metastasis in OSCC. However, it is unclear as to whether CCL4 correlates with VEGF-C expression and lymphangiogenesis in OSCC. We found that CCL4 increased VEGF-C expression and promoted lymphangiogenesis in oral cancer cells *in vitro* and *in vivo*. miR-195-3p mimic reversed CCL4-enhanced VEGF-C expression. CCL4 stimulation of oral cancer cells augmented JAK2 and STAT3 phosphorylation. Thus, CCL4 may be a new molecular therapeutic target for inhibition of lymphangiogenesis and metastasis in OSCC.

## Introduction

Oral squamous cell carcinoma (OSCC) is the most common malignant tumor of the head and neck (~95%) in Taiwan, accounting for the fourth highest incidence of cancers in the male population and the first cause of death among 40-year-old Taiwanese men (1). As many as 50% or more of Taiwanese patients with OSCC present with stage III or stage IV at diagnosis, leading to a low overall 5-year survival rate (2). This emphasizes the need to detect OSCC as early as possible and identify the mechanisms driving tumor lymphangiogenesis to improve survival. Chemokine (C–C motif) ligand 4, also known as CCL4, or macrophage inflammatory protein-1 (MIP-1β), plays a key role in inflammation and immune regulation, as well as cancer progression (3, 4). Recent evidence suggests that tumor cell-derived CCL4 promotes breast cancer metastasis by inducing CCR5-expressing fibroblasts to express connective tissue growth factor/CCN2 (5). Serum concentrations of CCL4 have been found to be significantly higher in patients with head and neck squamous cell carcinoma compared with controls (6), and we have previously reported that CCL4 polymorphisms may enhance susceptibility to oral cancer (7). This study set out to determine the role of CCL4 in OSCC.

Metastasis is a leading cause of cancer-related death. Important steps in metastasis are thought to involve lymphangiogenesis (the formation of new lymphatic vessels) and the remodeling of existing lymphatics (8). Tumors can actively promote lymphangiogenesis and lymphatic enlargement, and in several cancers, higher numbers of lymphatic vessels correlate closely with metastasis and clinical outcome (9–11). Inhibition of cancer-mediated lymphangiogenesis has therefore been considered to be an effective means of preventing the spread of cancer (12). Lymphangiogenic growth factors, in particular vascular endothelial growth factor C (VEGF-C), are derived from tumor cells and promote tumor lymphangiogenesis by activating the endothelial receptor tyrosine kinases VEGFR2 and VEGFR3 (13). VEGF-C acts directly on lymphatic endothelial cells (LECs) to induce survival, proliferation, migration, and tube formation (14). VEGF-C/VEGFR3 expression is associated with lymph node metastasis in OSCC (15). It is known that various signaling systems influence tumor lymphangiogenesis through the modulation of VEGF-C. For instance, JAK/STAT3 signaling is associated with oral cancer cell proliferation, invasion and angiogenesis (16), while STAT3 signaling induces LEC migration and tube formation (17).

MicroRNAs (miRNAs), a class of small, non-coding RNA molecules measuring about 18–22 nucleotides in length, regulate gene expression by directly targeting the 3′-untranslated region (3′-UTR) of their target mRNAs (18, 19). miRNAs regulate tumor metastasis by altering cancer cell proliferation and migration and are involved in the formation and functioning of different microenvironments (20). Findings suggest that the deregulated expression of miRNAs may modulate tumor angiogenesis and lymphangiogenesis through the targeting of VEGF-C (21). In non-small cell lung cancer, miR-195 reportedly suppresses cell proliferation, migration and invasion by directly inhibiting MYB expression (22). Notably, aberrant miRNA expression associated with tumor progression, nodal metastasis, and blood vessel density has been observed in OSCC (23). We wanted to further clarify how miRNAs regulate CCL4-mediated VEGF-C expression in OSCC. In this study, we show that CCL4 induces VEGF-C expression in OSCC by activating the JAK2/STAT3 signaling pathway. By contrast, miR-195-3p interrupts CCL4-induced VEGF-C expression, which subsequently enhances lymphangiogenesis in LECs.

## Materials and Methods

### Subjects

During 2014–2016, we recruited 154 newly diagnosed patients with OSCC scheduled to undergo surgery in China Medical University Hospital, Taichung, Taiwan. Clinicopathological data regarding age, classification of tumor, lymph node, metastasis (TNM) staging, and pathological features were collected from medical records. Patients were clinically staged according to the American Joint Committee on Cancer TNM system. The study also included 11 healthy participants without any previous history of cancer. Recruitment of patients into this study was approved by the Institutional Review Board of China Medical University Hospital. The study was granted research access to surgically excised OSCC specimens matched with non-tumor epithelial tissues from each patient.

### Materials

Rabbit polyclonal lymphatic vessel endothelial hyaluronan receptor 1 (LYVE-1) antibody was purchased from Abcam. Santa Cruz Biotechnology supplied the following rabbit polyclonal antibodies: JAK2, VEGF-C, CCL4, and CCR5, as well as β-actin diluted 1:3,000 and STAT3-specific mouse monoclonal antibodies (mAbs) diluted 1:1,000. Recombinant human CCL4 and VEGF-C were purchased from PeproTech. Recombinant human CCL4 contains the substitutions of histidine for arginine at the 22nd position of the sequence and of glycine for serine at the 47th position. Staff from PeproTech tested the biological activity of recombinant human CCL4 for its ability to chemoattract human blood monocytes. This testing confirmed the biological activity of CCL4 and showed that this product has equivalent biological activity as a natural chemokine. Inhibitors of JAK2 (product ID: CAS 457081037) and STAT3 (product ID: C1889) were purchased from Calbiochem (San Diego, CA, USA). A TRIzol kit was purchased from MDBio Inc., and TaqMan one-step PCR Master Mix was purchased from Applied Biosystems. Dulbecco’s modified Eagle’s medium (DMEM) were purchased from Gibco-BRL Life Technologies. miR-195-3p mimic and control miRNA were purchased from Invitrogen. Thermo Fisher Scientific Inc. provided the Thermo Scientific Pierce BCA Protein Assay Kit. All other chemicals were purchased from Sigma-Aldrich.

### Cell Culture

The human OSCC cell line SAS was kindly provided by Dr. Shun-Fa Yang (Chung Shan Medical University). Human OSCC cell line SCC4 was provided from Bioresource Collection and Research Center. OSCC cells were cultured at 37°C and 5% CO_2_ in DMEM/F-12 medium containing penicillin, streptomycin and 10% FBS.

Human LEC was provided from Lonza and seeded onto 1% gelatin-coated plastic and grown in an EGM-2 BulletKit containing EBM-2 basal medium and a SingleQuots kit.

### Patient Serum and Tissue Preparation

All serum and tissue samples were collected from patients with OSCC undergoing surgical resection in China Medical University Hospital. Written informed consent was obtained from each study participant before enrollment. This study was approved by the Institutional Review Board of China Medical University Hospital (CMUH105-REC3-042).

### ELISA

SAS and SCC4 cells were cultured in 6-well plates until they reached confluence, then switched to serum-free medium and treated with CCL4 for 24 h, pharmacological inhibitors, or transfected with miR-195-3p mimic, then stimulated with CCL4 for another 24 h. The conditioned medium (CM) was collected after 24 h and stored at −80°C.

A VEGF-C ELISA kit (PeproTech, Rocky Hill, NJ, USA) assayed VEGF-C protein concentrations in CM and patient serum.

### Tube Formation Assay

Matrigel was added in concentrations of 100 µL/well into 48-well plates, which were incubated at 37°C for 30 min. Following gel formation, LECs (1 × 10^4^ cells) were seeded into each well on a layer of polymerized Matrigel in cultured media containing EGM-2MV BulletKit Medium complete medium (50%) and CM (50%) containing with or without CCR5 and VEGF-C antibodies, then incubated for 6 h at 37°C. Tube formation was imaged using an inverted phase-contrast microscope. MacBiophotonics Image J software calculated tube branches and total tube lengths.

### Migration Assay

The migration assay used Transwell cell culture chambers with a 24-well 8.0 µm pore size. LECs (1 × 10^4^ cells/well) were seeded onto the upper chamber with 0% EGM-2 BulletKit medium, incubated in the lower chamber with 50% EGM-2 BulletKit complete medium and 50% CM containing with or without CCR5 and VEGF-C antibodies, then incubated for 24 h. The cells in the upper chamber were then fixed in 4% formaldehyde solution for 30 min and stained for 30 min with 0.05% crystal violet. Cotton-tipped swabs were used to remove cells on the upper side of the filters, and the filters underwent three PBS washes. We used an inverted phase-contrast microscope to measure cell migration, by counting the number of stained cells in three random fields.

### Quantitative Real-time PCR

A TRIzol kit extracted total RNA; 2 µg was reverse transcribed into cDNA. TaqMan one-step PCR Master Mix was used for quantitative real-time polymerase chain reaction (q-PCR) analysis. Total complementary DNA (100 ng/25 μL reaction) was mixed with sequence-specific primers. The cycling conditions of q-PCR assays conditions consisted of 10 min of polymerase activation at 95°C, followed by 40 cycles at 95°C for 15 s and 60°C for 60 s and detected by using a StepOnePlus sequence detection system. Normalization of gene expression data used endogenous glyceraldehyde 3-phosphate dehydrogenase as the internal control for mRNA. Gene expression was normalized for relative mRNA in normal healthy tissue and served as the normal control.

For miRNA, Mir-X™ miRNA First-Strand Synthesis was used by reverse transcription. The specific primer of miR-195-3p was as follows: 5′-CCAATATTGGCTGTGCTGCTCC-3′. U6 snRNA levels were used for normalization.

### Western Blot Analysis

The BCA Protein Assay Kit determined concentrations for each sample protein. SDS-PAGE gels were used for separating protein samples, and then proteins were transferred to immobilon polyvinyl difluoride membranes. Membranes were blocked with 5% BSA for 1 h at room temperature then washed three times in Tris-buffered saline with 0.05% Tween 20. Before incubation with peroxidase-conjugated secondary antibody for 1 h, membranes were incubated with antibody specific for JAK and STAT for 2 h. Blots were visualized using enhanced chemiluminescence and imaged on Kodak X-OMAT LS film.

### Luciferase Activity Assay

Cells were transfected with VEGF-C 3′-UTR luciferase plasmids by Lipofectamine 2000. Twenty-four hours later, cells were collected and lysed with reporter lysis buffer. The Dual-Luciferase^®^ Reporter Assay System protocol measured luciferase and Renilla activities in the cellular extracts. We calculated relative luciferase activity according to the ratio of luciferase/Renilla activity and normalized it to control cell activity.

### *In Vivo* Mouse Model

All animal experiments followed protocols issued by the China Medical University (Taichung, Taiwan) Institutional Animal Care and Use Committee. Male BALB/c nude mice (5 weeks old) were randomly divided into two groups: SAS/control-shRNA or SAS/CCL4-shRNA. These cells were injected at a dose of 1 × 10^3^ cells into the oropharynx of each animal. After 12 days, mice were euthanized by CO_2_ inhalation. Subsequently, the tumor cells were removed and photographed, photographed, fixed in 10% formalin and embedded in paraffin, then subjected to immunohistochemical (IHC) staining with LYVE-1, CCL4, and VEGF-C.

### IHC Staining

Tumor cell sections were deparaffinized and rehydrated. Endogenous peroxidase activity was blocked in methanol for 10 min with 3% hydrogen peroxide. Antigen retrieval was carried out for all sections in pH 6 sodium citrate buffer 0.01 M at 95°C for 25 min. Human LYVE-1, CCL4 or VEGF-C antibody was applied at a dilution of 1:200 then incubated 2 h. Antibody binding signals were detected with the NovoLink Polymer Detection System (Leica Microsystems) and visualized with the diaminobenzidine reaction. We used Image J software to obtain IHC data, which were semi-quantitatively scored by calculating intensity of the staining and the percentage of positive detection, whereby the results were very low (score of 1: 0–25% of positive area), or weakly (score of 2: 25–50%), moderately (score of 3: 50–75%), or strongly positive (score of 4: 75–100%).

### Statistics

Pearson’s chi-squared or Fisher’s exact test was used to compare differences between low and high CCL4 expression. Specifically, Fisher’s exact test was used when the expected value in any cell of a contingency table was below 5. Correlation analysis was conducted using the Pearson correlation coefficient test or Spearman’s rank correlation coefficient test. Data are expressed as the mean ± SE. Between-group differences were analyzed using the Student’s *t*-test of variance. All differences were considered significant if the *p* value was less than 0.05.

## Results

### Clinical Significance of CCL4 and VEGF-C Expression in OSCC

**Figure 1:**
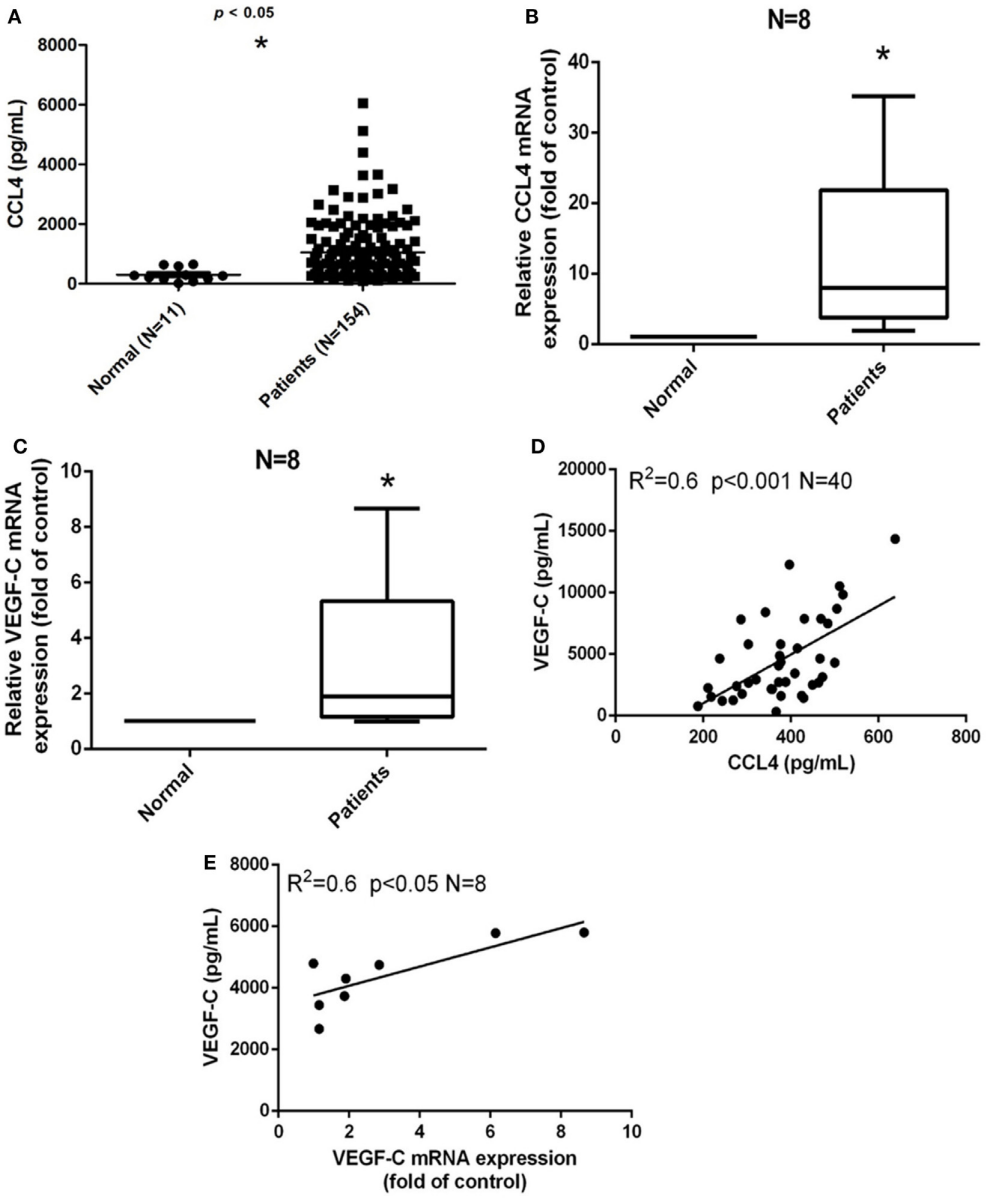
Clinical significance of CCL4 and vascular endothelial growth factor C (VEGF-C) expression in oral squamous cell carcinoma (OSCC). **(A)** Serum CCL4 levels in patients with OSCC. ELISAs were performed to quantify CCL4 levels in serum from (•) 11 normal, healthy volunteers and from (▪) 154 patients diagnosed with OSCC. **(B,C)** CCL4 and VEGF-C mRNA expression was detected by qRT-PCR in eight paired tissue samples. **(D)** ELISAs of serum from eight patients with OSCC revealed a positive correlation between serum VEGF-C and CCL4 (Pearson’s correlation coefficient, *R*^2^ = 0.6) and **(E)** a positive correlation between VEGF-C and mRNA expression (Spearman’s rank correlation coefficient, *R*^2^ = 0.6). Each experiment was repeated three times (*N* = 3). *p < 0.05 when compared with controls.

Our previous study demonstrated the involvement of CCL4 gene polymorphisms in oral cancer development (7). We have previously reported that CCL4 expression is higher in OSCC patients than in healthy individuals (controls) (7). Similarly, in this investigation, we found higher levels of CCL4 expression in the 154 patients with OSCC as compared with controls (Figure 1A). Univariate analysis revealed that higher pathological T and N status was significantly correlated with higher CCL4 expression (Table 1). Therefore, we thought that CCL4 might have an important role in oral cancer progression and lymph node metastasis. Metastasis to lymph nodes is largely responsible for the progression and dissemination of head and neck carcinoma; VEGF-C is the most studied growth factor associated with lymphangiogenesis (24). We have previously demonstrated higher levels of VEGF-C expression in tumor specimens than in normal tissues (25). Likewise, this study revealed higher levels of CCL4 and VEGF-C mRNA expression in OSCC tissue compared with tissue from healthy controls, indicating the important role of CCL4 expression in VEGF-C expression and in OSCC lymph node metastasis (Figures 1B,C). In addition, we observed a significant correlation between CCL4 and VEGF-C levels in serum samples as well as a significant correlation between VEGF-C mRNA expression and circulating VEGF-C in tumor specimens (Figures 1D,E). These findings indicate a positive correlation between CCL4 and VEGF-C levels in human OSCC.

**Table 1:** Correlation of clinical status with CCL4 expression [ranging from low (0–25%) to high (75–100%)] of 154 oral cancer patients.

Variable	Low CCL4 (*n* = 35)	High CCL4 (*n* = 37)	*p* Value
**Age (years)**			
<50	10	6	
>50	25	31	*p* = 0.208
**Clinical T status**			
T1 + T2	18	14	
T3 + T4	17	23	*p* = 0.246
**Clinical N status**			
N0 + N1	22	22	
N2 + N3	13	15	*p* = 0.768
**Clinical stage**			
I + II	11	11	
III + IV	24	26	*p* = 0.876
**Pathological T status**			
T1 + T2	25	17	
T3 + T4	10	20	*p* = 0.028*
**Pathological N status**			
N0 + N1	32	23	
N2 + N3	3	14	*p* = 0.003*
**Pathological stage**			
I + II	24	18	
III + IV	11	19	*p* = 0.087

*Pearson’s chi-square statistics and Fisher’s exact test were used to define low and high levels of CCL4 expression*.

*p Value < 0.05 as statistically significant.

### CCL4 Promotes VEGF-C-Dependent Lymphangiogenesis in Human OSCC Cells

**Figure 2:**
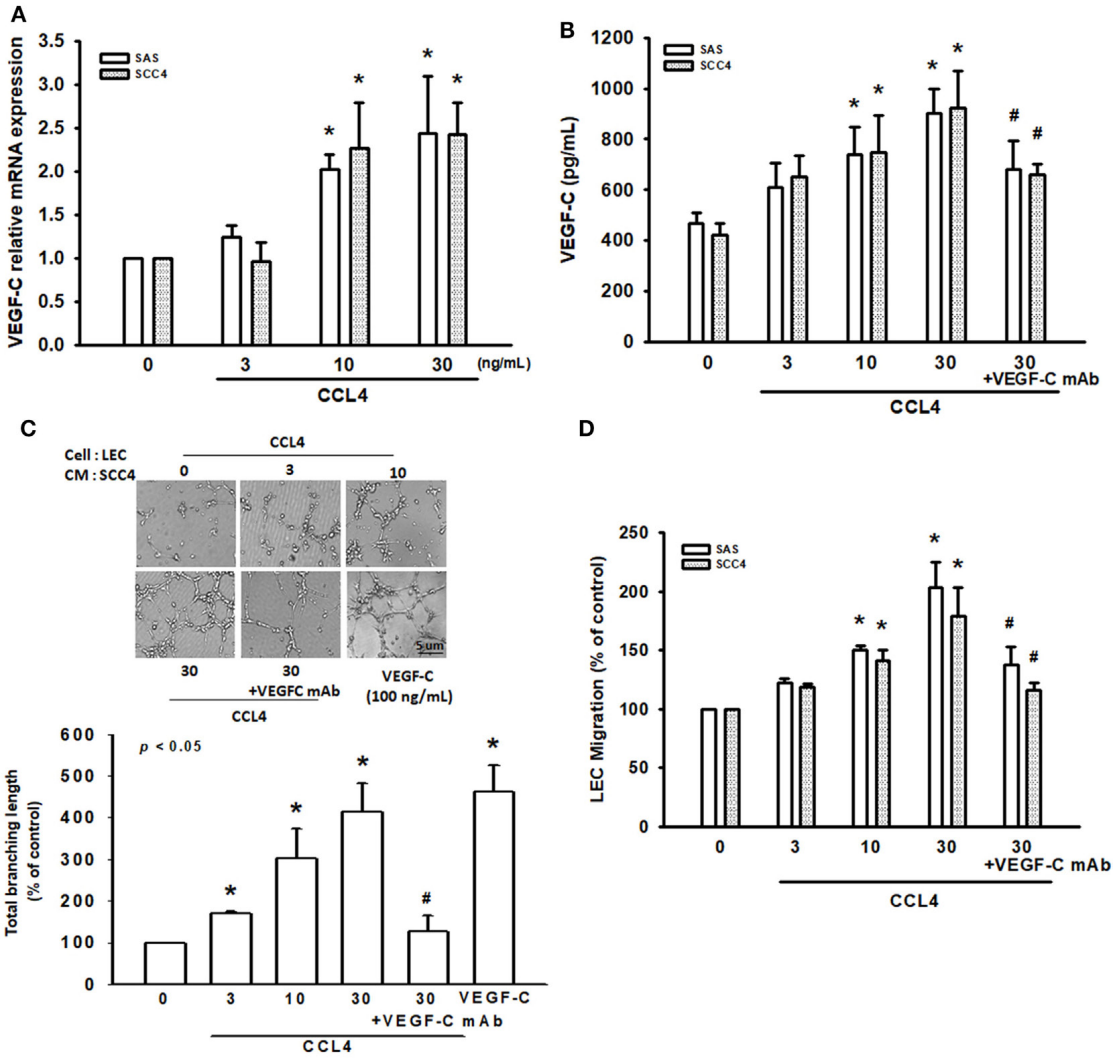
CCL4 promotes vascular endothelial growth factor C (VEGF-C)-dependent lymphangiogenesis in human oral squamous cell carcinoma cells. **(A,B)** Cells were treated with various concentrations of CCL4 (0–30 ng/mL), and the mRNA and protein expressions were detected by qRT-PCR and ELISA. **(C,D)** Cells were treated with various concentrations of CCL4 (0–30 ng/mL). Culture medium was collected as conditioned medium and applied to lymphatic endothelial cells (LECs) for 24 h. LEC capillary-like structure formation and cell migration were examined by tube formation assay and the Transwell migration assay, respectively. Each experiment was performed three times (*N* = 3). *p < 0.05 when compared with control. ^#^*p* < 0.05 when compared with the group treated with CCL4 (30 ng/mL).

Vascular endothelial growth factor C activation reportedly regulates OSCC lymphangiogenesis (26). To determine whether VEGF-C plays a role in CCL4-induced lymphangiogenesis of OSCC cells, we incubated two OSCC cell lines (SCC4 and SAS cells) with human recombinant CCL4. Whereas CCL4-induced significant increases in VEGF-C mRNA and protein secretion (Figures 2A,B), LEC proliferation was unaffected (Figure S1 in Supplementary Material). As with previous research showing that LEC proliferation, migration and tube formation generates new lymphatic vessels essential for tumor lymphangiogenesis (8), we found that incubating LECs for 24 h with CM from CCL4-treated OSCC cells dramatically promoted LEC tube formation and migration (Figures 2C,D). Similar results were obtained after performing the migration assay for 16 h (Figure S2 in Supplementary Material). Notably, VEGF-C mAb abolished these activities (Figures 2C,D), which implies that CCL4 promotes lymphangiogenesis *via* a VEGF-C-dependent pathway.

### CCL4 Promotes VEGF-C Expression and Lymphangiogenesis *via* the CCR5 Receptor

**Figure 3:**
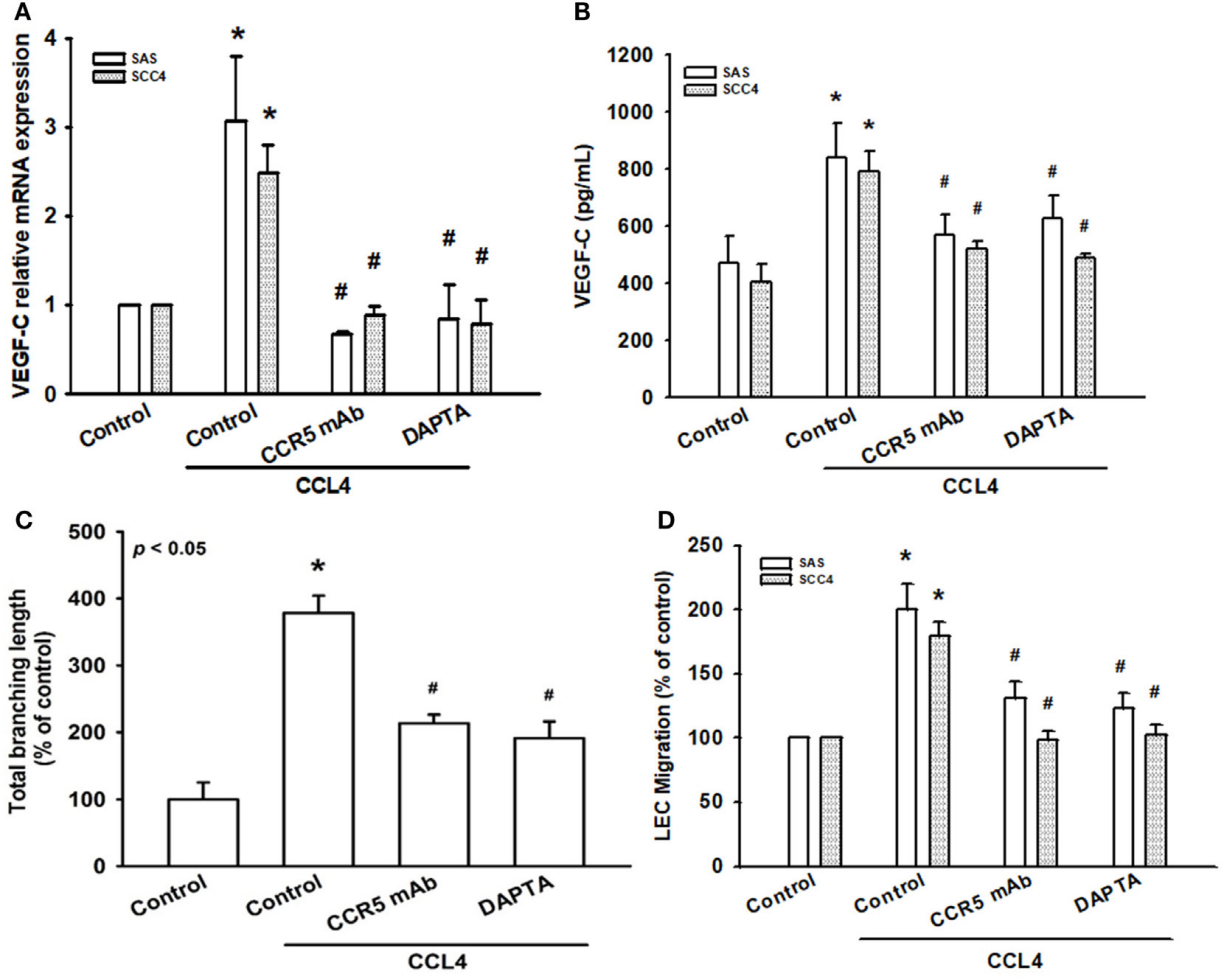
CCL4 promotes vascular endothelial growth factor C (VEGF-C) expression and lymphangiogenesis *via* the CCR5 receptor. Cells were pretreated with CCR5 monoclonal antibody (mAb) (5 μg/mL) or DAPTA (1 nM) for 30 min, then treated with CCL4 (30 ng/mL) for 24 h. **(A,B)** VEGF-C mRNA and protein expression was detected by qRT-PCR and ELISA. **(C,D)** Culture medium was collected as conditioned medium and applied to lymphatic endothelial cells (LECs) for 24 h. LEC capillary-like structure formation and cell migration were examined by tube formation assay and the Transwell migration assay, respectively. Each experiment was performed three times (*N* = 3). *p < 0.05 when compared with controls. ^#^*p* < 0.05 when compared with the CCL4-treated group.

CCL4 has specificity for the CCR5 receptor (27). To clarify the interaction between CCL4 and CCR5, we used the selective CCR5 mAb and the CCR5 antagonist DAPTA. CCL4-induced VEGF-C mRNA expression was markedly attenuated by the CCR5-specific mAb and DAPTA, which was confirmed at a protein level (Figures 3A,B). We then used an *in vitro* LEC model to investigate whether CCL4-dependent VEGF-C expression induces lymphangiogenesis *via* the CCR5 receptor. Similarly, DAPTA and CCR5 mAb notably inhibited tube formation and migration in human LECs (Figures 3C,D). It appears that VEGF-C production and lymphangiogenesis in human OSCC cells is enhanced by the CCL4/CCR5 axis.

### JAK2 and STAT3 Activation Are Involved in CCL4-Induced Promotion of VEGF-C Expression and Lymphangiogenesis

**Figure 4:**
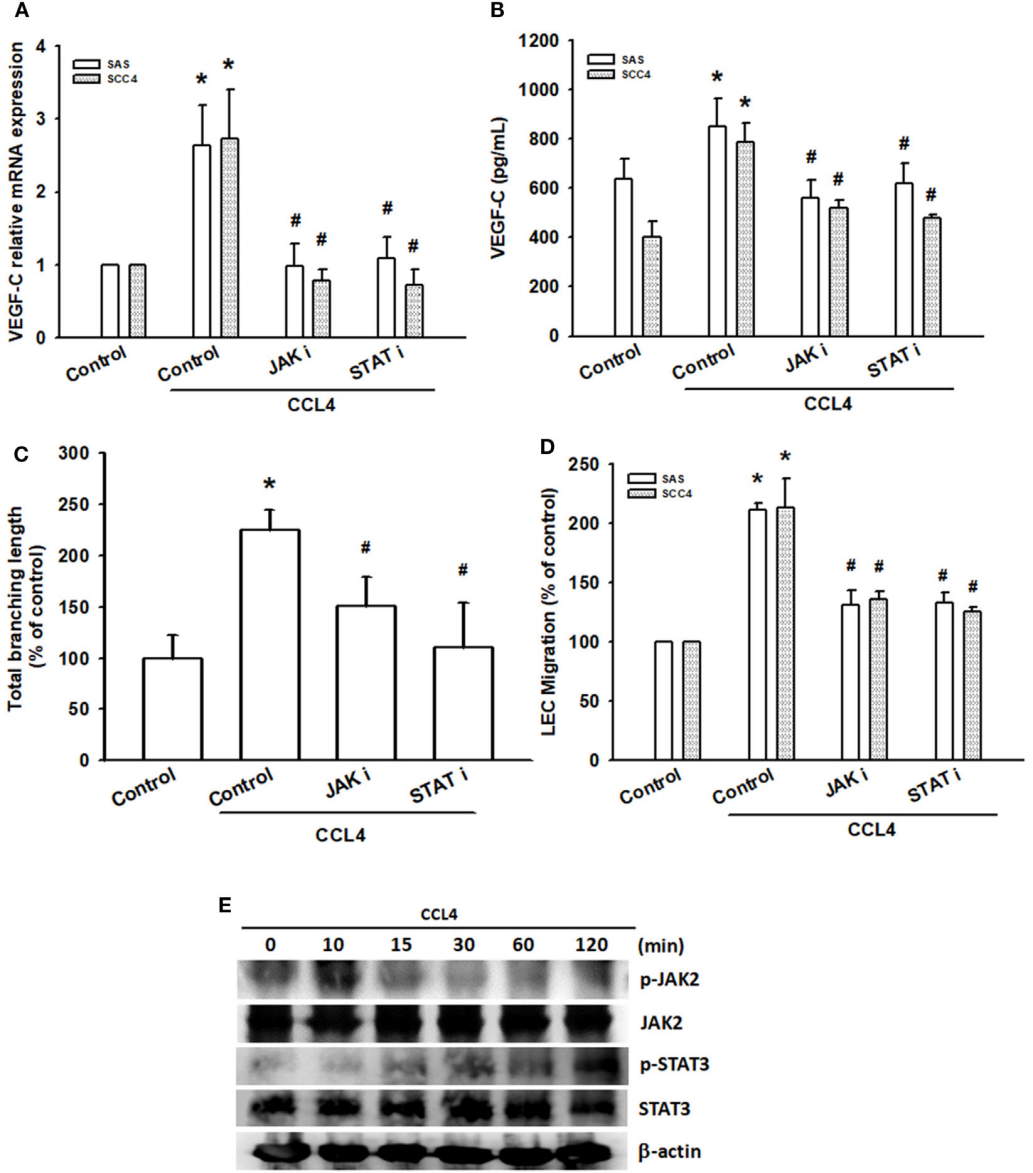
JAK2 and STAT3 activation are involved in CCL4-induced promotion of vascular endothelial growth factor C (VEGF-C) expression and lymphangiogenesis. Cells were pretreated with a JAK2 inhibitor (JAK i; 1 nM) or STAT3 inhibitor (STAT i; 10 µM) for 30 min, before treatment with CCL4 (30 ng/mL) for 24 h. **(A,B)** VEGF-C mRNA and protein expression was detected by qRT-PCR and ELISA. **(C,D)** Culture medium was collected as conditioned medium and applied to lymphatic endothelial cells (LECs) for 24 h. LEC capillary-like structure formation and cell migration were examined by tube formation assay and the Transwell migration assay, respectively. **(E)** Cells were incubated with CCL4 (30 ng/mL) for the indicated times; JAK2 and STAT3 phosphorylation was detected by Western blot (molecular weight). Each experiment was performed three times (*N* = 3). *p < 0.05 when compared with controls. ^#^*p* < 0.05 when compared with the CCL4-treated group.

The JAK2/STAT3 signaling pathway is implicated in cell invasion and angiogenesis in oral cancer (16), as well as head and neck metastasis (16, 28). We analyzed this pathway in CCL4-induced VEGF-C expression and lymphangiogenesis in OSCC cells. Pretreatment with a JAK2-specific inhibitor (JAK i) significantly reduced exogenous CCL4-induced VEGF-C expression (Figures 4A,B), as well as LEC tube formation and migration (Figures 4C,D). In addition, following CCL4 stimulation, JAK2 activity and phosphorylation increased in a time-dependent manner (Figure 4E). Thus, CCL4 appears to act *via* the JAK2 signaling pathway to promote VEGF-C expression and lymphangiogenesis in OSCC cells.

Activated JAK2, phosphorylated STAT3, resulting in translocation of activated STAT3 dimers to the nucleus (29). In the nucleus, STA-3 induces various cellular processes that promote cancer progression (30). Similarly, pretreatment with the STAT3 inhibitor (STAT i) reduced CCL4-mediated VEGF-C expression, LEC tube formation and migration (Figures 4A–D). In addition, CCL4 significantly increased phosphorylation of STAT3 (Figure 4E). CCL4 appears to enhance VEGF-C expression and lymphangiogenesis in OSCC cells through the JAK2/STAT3 signaling pathway.

### CCL4 Promotes VEGF-C Expression and Lymphangiogenesis by Downregulating miR-195-3p Expression

**Figure 5:**
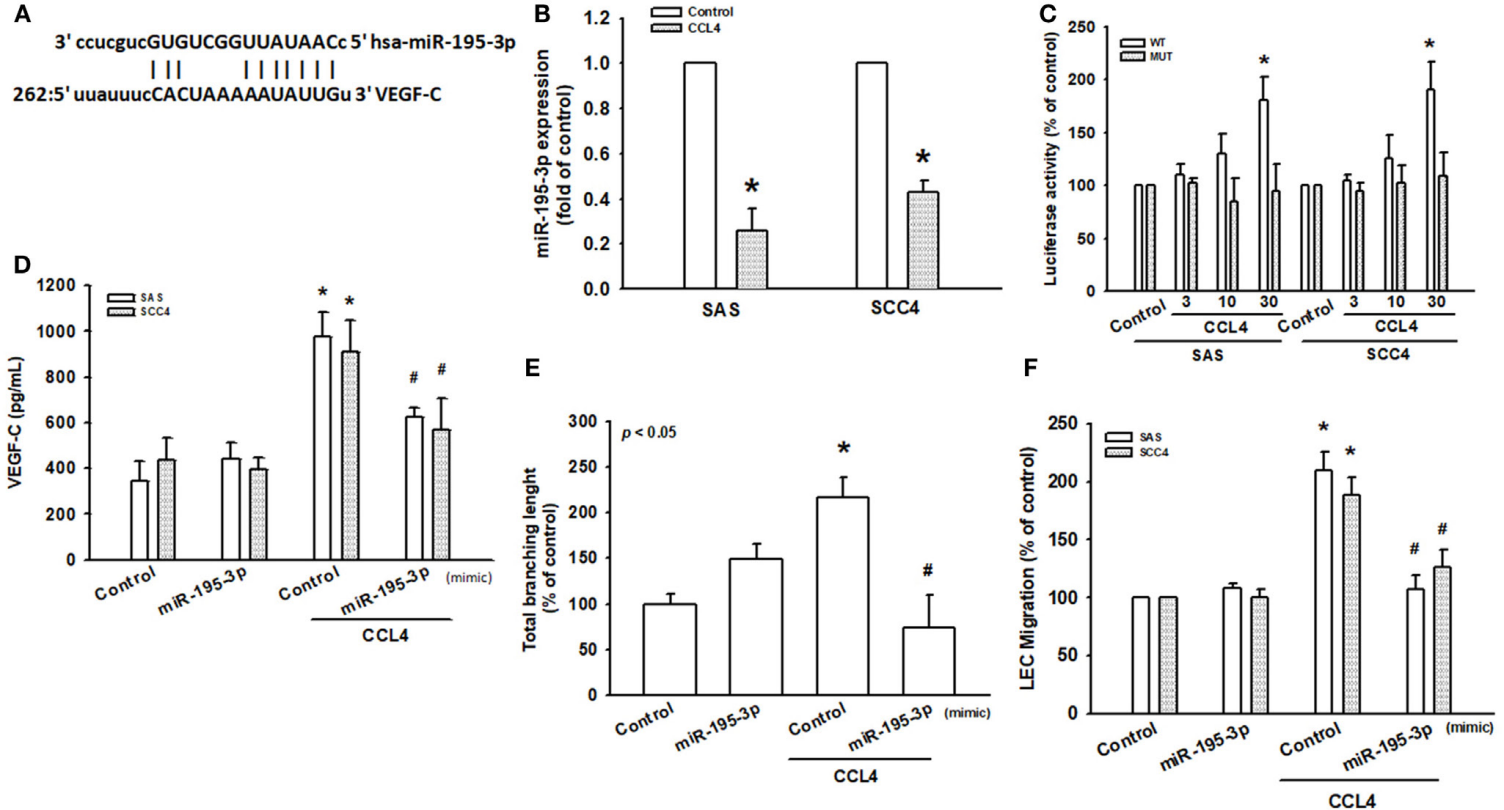
CCL4 promotes vascular endothelial growth factor C (VEGF-C) expression and lymphangiogenesis by downregulating miR-195-3p expression. **(A)** Schematic 3′-untranslated region (3′-UTR) representation of human VEGF-C containing the miR-195-3p binding site. **(B)** Cells were stimulated with CCL4 (30 ng/mL), and microRNA expression was examined by qRT-PCR. **(C)** Cells were transfected with 3′-UTR reporter assay plasmids for 24 h, then incubated with CCL4 (0–30 ng/mL), and relative luciferase activity was measured. **(D)** Cells were transfected with control or miR-195-3p mimic for 24 h, then incubated with CCL4 (30 ng/mL). VEGF-C protein expression was detected by ELISA. **(E,F)** Conditioned medium was applied to lymphatic endothelial cells (LECs) for 24 h. LEC capillary-like structure formation and cell migration were examined by tube formation assay and the Transwell migration assay, respectively. Each experiment was performed three times (*N* = 3). *p < 0.05 when compared with controls. ^#^*p* < 0.05 when compared with the CCL4-treated group.

Recent studies have reported that miRNAs function as modulators of VEGF-C and tumor lymphangiogenesis (31, 32). Using TargetScan (www.TargetScan.org and www.microrna.org), we found that the 3′-UTR of VEGF-C mRNA harbors potential binding sites for miR-195-3p (Figure 5A). Moreover, we found that CCL4 downregulated miR-195-3p expression (Figure 5B). To explore whether miR-195-3p regulates the 3′-UTR of VEGF-C, we constructed luciferase reporter vectors harboring either the wild-type 3′-UTR of VEGF-C mRNA (WT VEGF-C-3′-UTR) or mismatches in the predicted miR-195-3p binding site (MUT VEGF-C-3′-UTR) and transfected them into both SAS and SCC4 cell lines. We found that CCL4 increased luciferase activity in the WT plasmid but not in the MUT plasmid (Figure 5C). We hypothesized that miR-195-3p mediates CCL4-promoted VEGF-C dependent lymphangiogenesis in human OSCCs. Indeed, transfection with miR-195-3p mimic reduced CCL4-induced VEGF-C expression, LEC tube formation and migration (Figures 5D–F). miR-195-3p appears to suppress VEGF-C protein expression *via* binding to the 3′-UTR of the human *VEGF-C* gene.

### Inhibiting CCL4 Expression Suppresses Lymphangiogenesis *In Vivo*

**Figure 6:**
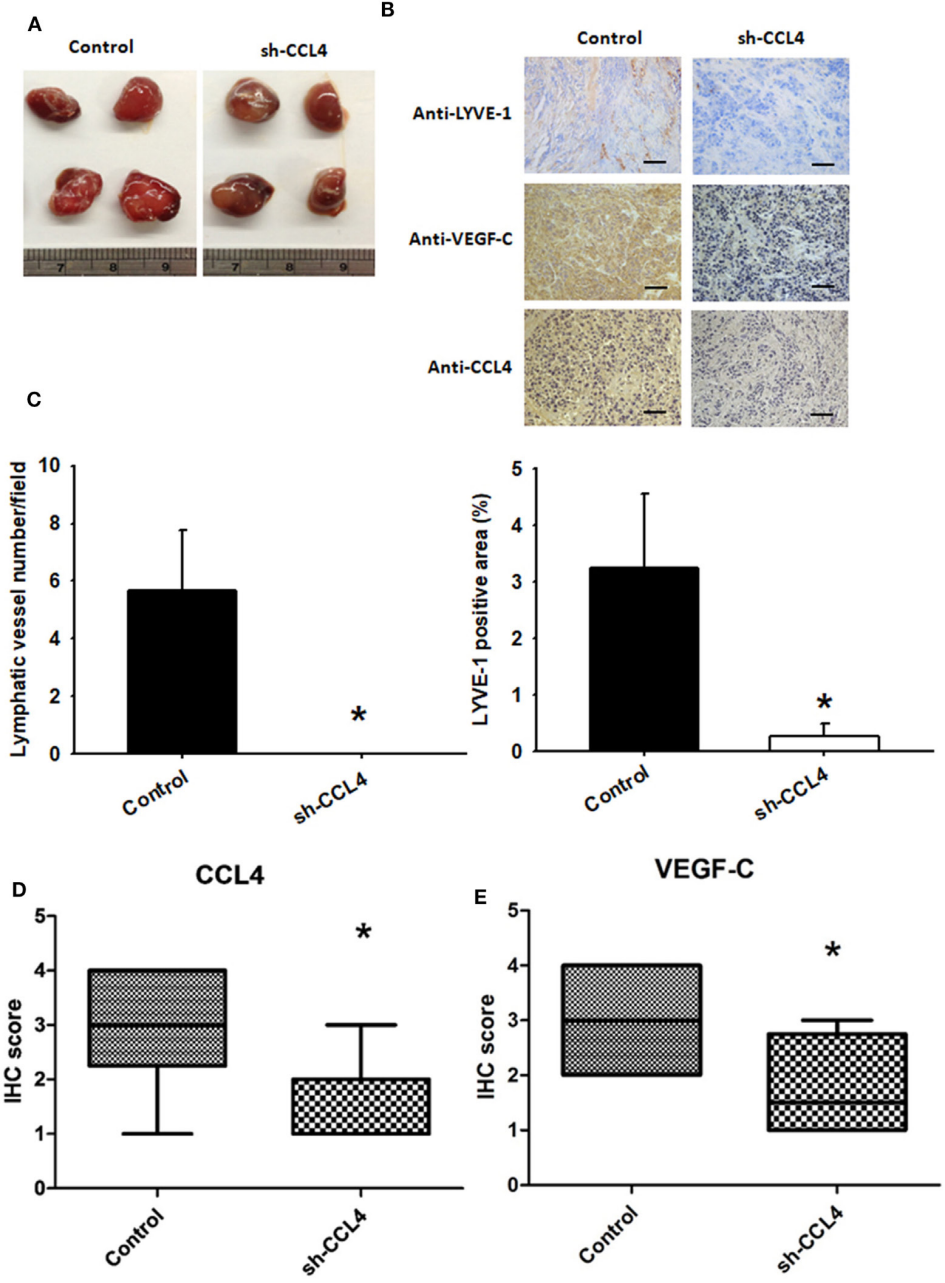
Inhibiting CCL4 expression suppresses lymphangiogenesis *in vivo* SAS cells stably expressing control (*n* = 12) or CCL4-shRNA (*n* = 12) were established. **(A)** After 12 days, the mice were sacrificed, and the tumors were excised and photographed (*N* = 12). **(B–E)** Immunohistochemical analysis of LYVE-1 (*n* = 3), CCL4 (*n* = 3), and vascular endothelial growth factor C (VEGF-C) (*n* = 3) expression in tumors (bar = 50 μm). *p < 0.05 when compared with controls.

*In vitro* data demonstrated that transfecting human OSCC cells with CCL4-shRNA suppressed LEC tube formation and migration (Figure S4 in Supplementary Material). We next investigated the role of CCL4 *in vivo*. SAS cells stably expressing CCL4-shRNA were used to inoculate nude mice. On the 12th day after inoculation, the final mean tumor weights and volumes (Figure S3 in Supplementary Material) did not differ between controls and sh-CCL4-treated mice (Figure 6A). To examine CCL4-associated lymphangiogenesis, we used LYVE-1, a lymph-specific receptor for hyaluronan and a homolog of CD44. IHC analysis revealed that CCL4 knockdown reduced the number of lymphatic vessels stained for anti-LYVE-1 (Figure 6B). IHC staining of murine tissue revealed substantially lower levels of LYVE-1 expression from sh-CCL4 mice compared with controls. We also found that the number of lymphatic vessels and LYVE-1 positive areas were reduced by knockdown of CCL4 (Figures 6B,C). Similarly, sh-CCL4 mice had lower levels of CCL4 and VEGF-C tissue expression compared with control mice (Figures 6B,D,E). The results suggest that CCL4 promotes VEGF-C dependent tumor-associated lymphangiogenesis *in vivo*.

## Discussion

Oral squamous cell carcinoma with lymph node metastasis indicates a poor outcome and a higher risk for recurrence or metastasis (33). Lymphangiogenesis is a critical step in tumor invasion and metastasis. VEGF-C-induced tumor lymphangiogenesis enhances the growth of lymphatic sprouts and contributes to the metastatic process (8, 34). Understanding the underlying mechanisms of tumor lymphangiogenesis has the potential to lead to therapeutic approaches inhibiting metastatic spread. The essential role played by CCL4 in breast cancer metastasis has been reported in a previous study (5). Furthermore, evidence suggests that CCL4 secreted by monocytic myeloid-derived suppressor cells (MDSCs) may promote tumor growth by attracting tumor-infiltrating regulatory T cells (35), and that the network of MDSCs and circulating tumor cells stimulates distal metastasis by avoiding immune surveillance (36). Our study used BALB/c nude mice, which lack a thymus to produce mature T-cells and suffer from a lack of cell-mediated immunity, making them appropriate for investigating tumor growth and metastasis. On the other hand, this animal model is not suitable for investigating tumor inflammation and immune cell infiltration. More appropriate tumor implantation models are needed to investigate the role of CCL4 in OSCC. Our study yields useful insights into the role of CCL4 in OSCC lymphangiogenesis. We found that higher CCL4 expression levels were associated with higher T and N status in OSCC. We also observed that CCL4 increases VEGF-C production by downregulating miR-195-3p *via* the JAK2 and STAT3 signaling pathways in OSCC cells, which indicates that CCL4 could serve as a novel therapeutic target in OSCC lymphangiogenesis.

CCL4L, a non-allelic copy of chemokine CCL4, differs in its protein by just a single amino acid (37). CCL4/CCL4L is known to promote immune cell infiltration in psoriasis, including T helper type-1 cells, regulatory T cells, monocytes, and dendritic cells (38). Two main allelic variants of CCL4L exist: the originally described variant, CCL4L1, and a second allelic variant with a nucleotide change in the intron 2 acceptor splice site, CCL4L2 (39). CCL4L gene copy number variation is known to modify susceptibility to or control of HIV-1 infection (40). Although CCL4L is associated with inflammation and infection, no evidence exists as to a relationship between CCL4L and tumor lymphangiogenesis. Further research is needed to assess the complementary effect of CCL4L and its copies on cancer progression and metastasis.

Recent evidence reveals the involvement of chemokines and their specific receptors in tumor lymphangiogenesis. For instance, the C–C chemokine receptor type 7 (CCR7), located mainly on the membrane of mature dendritic and T cells, is capable of interacting with its specific ligand CCL21 to facilitate lymph node metastasis in esophageal cancer (41). Moreover, CCL21/CCR7 signalings VEGF-C expression and secretion and promotes breast cancer-induced lymphangiogenesis *via* LEC activation (42). Clinicopathological data also show that chemokines and their receptors are involved in tumor lymphatic metastasis; for example, CCL21/CCR7 expression is related to poor outcomes in urinary bladder cancer (43). In this study, CM from CCL4-treated OSCC cells promoted tube formation and migration in LECs, indicating that CCL4 enhances lymphangiogenesis in OSCC cells.

CCR5 is a specific high-affinity receptor for CCL4 and plays a major role in cancer development by increasing inflammation and immune cell recruitment (44). In our study, CCR5 knockdown dramatically suppressed VEGF-C-induced lymphangiogenesis. We also showed that CCL4 and VEGF-C display a positive correlation in the OSCC xenograft model. CCL4 knockdown diminished the expression of lymphangiogenesis marker LYVE-1 and VEGF-C *in vivo*. Herein, we point out that CCL4/CCR5 axis induces VEGF-C-dependent lymphangiogenesis in human OSCC.

The JAK/STAT pathway is activated after chemokine receptor-mediated signaling (45). JAK2, a potential candidate signaling molecule, mediates chemokine-increased cell proliferation and migration (46). In this investigation, a JAK2 inhibitor antagonized CCL4-induced VEGF-C expression, while incubation of OSCC cells with CCL4-promoted JAK2 phosphorylation. This implies an essential role for JAK2 activation in CCL4-induced increases in VEGF-C production and lymphangiogenesis. STAT3 is considered to an important downstream transcription factor of JAK signaling (30). In this study, STAT3 inhibition reduced VEGF-C production, while CCL4-enhanced STAT3 phosphorylation. Thus, JAK2-dependent STAT3 activation may play a key role in CCL4-induced increases in VEGF-C expression and lymphangiogenesis.

MicroRNAs are small, non-coding RNA molecules of about 22 nucleotides that constitute a novel function as posttranscriptional gene regulators. They negatively regulate the expression of their target gene by binding to complementary 3′-UTR sequences of target mRNA (19, 47). miR-195-3p reportedly associates with renal cell carcinoma tumorigenesis, including cell proliferation, migration and invasion (48). Reduced miR-195-3p expression is associated with poor overall survival in patients with tongue squamous cell carcinoma (49), but its effect upon VEGF-C expression is unclear. We found that miR-195-3p, which harbors VEGF-C binding sites, was the most decreased miRNA after CCL4 stimulation (data not shown). This was supported by the finding that exogenous CCL4 reduced miR-195-3p expression, while co-transfection with a miR-195-3p mimic reduced CCL4-induced VEGF-C expression, as well as tube formation and migration in LECs. In addition, we found that miR-195-3p directly represses VEGF-C protein expression through binding to the 3′-UTR of the human VEGF-C gene, negatively regulating VEGF-C-mediated lymphangiogenesis.

Higher levels of CCL4 expression in serum from patients with OSCC are associated with more advanced T and N status. Our cellular experiments indicated that CCL4 promotes VEGF-C expression and lymphangiogenesis in OSCC. Not only does CCL4 induce VEGF-C expression and lymphangiogenesis by activation of the JAK2/STAT3 signaling pathways, but also, miR-195-3p inhibits CCL4-induced VEGF-C expression. We suggest that CCL4 deserves to be investigated as a molecular target in the inhibition of OSCC lymphangiogenesis and metastasis.

## Ethics Statement

All serum and tissue were collected from patients diagnosed with OSCC who had undergone surgical resection at China Medical University Hospital. All patients gave written consent before enrollment. This study was approved by the Institutional Review Board of China Medical University Hospital (CMUH105-REC3-042). All animal experiments were performed in accordance with a protocol approved by the China Medical University (Taichung, Taiwan) Institutional Animal Care and Use Committee.

## Author Contributions

M-YL had full access to all study data and assumes full responsibility for the integrity of the data and for the accuracy of its analysis. Study design—M-YL, H-CT, A-CC, and C-HT. Acquisition of data—M-YL, H-CT, M-HT, and C-HH. Analysis and interpretation of data—M-HT and C-HH. Manuscript preparation—M-YL, H-CT, and C-HT. Statistical analysis—C-HH and S-WW.

## Conflict of Interest Statement

The authors declare that the research was conducted in the absence of any commercial or financial relationships that could be construed as a potential conflict of interest.
