# Supplementary material for: Chemokine CCL4 Induces Vascular Endothelial Growth Factor C Expression and Lymphangiogenesis by miR-195-3p in Oral Squamous Cell Carcinoma
Source: Front Immunol. 2018 Mar 2;9:412. doi: 10.3389/fimmu.2018.00412 (PMC5863517; doi:10.3389/fimmu.2018.00412)
Supplement: Supplementary file 1 [file Image_1.PDF]

## Supplementary data

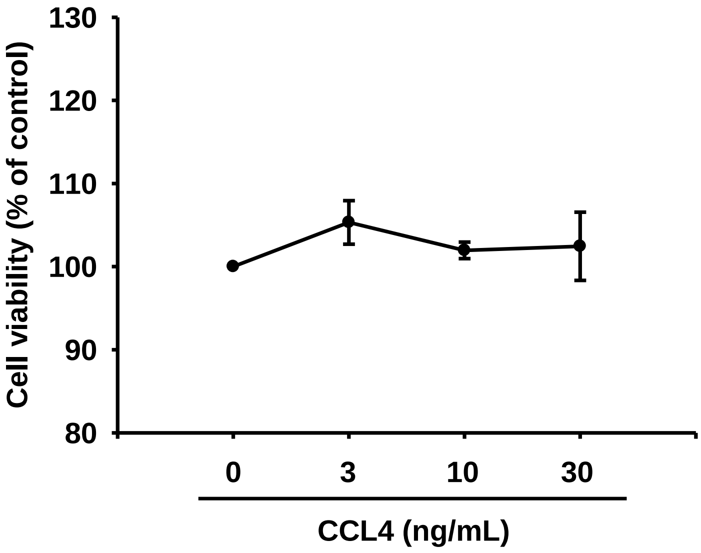


**Figure S1: Effect of CCL4 on LEC cell proliferation.** LEC cells were treated with various concentrations of CCL4 (0–30 ng/mL) for 24 h. Cell proliferation was detected by 3-(4,5-dimethylthiazol-2-yl)-2,5-diphenyltetrazolium bromide (MTT) assay. Each experiment was performed three times (N=3).

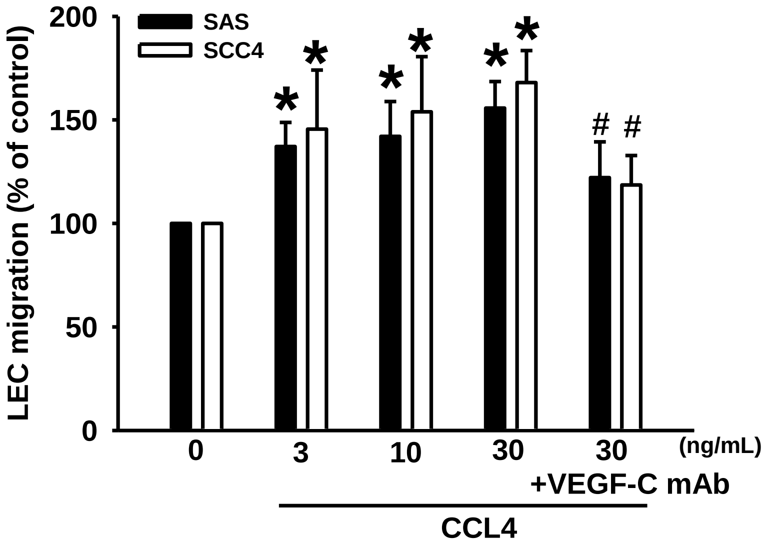


**Figure S2: CCL4 promotes LEC migration.** Cells were treated with various concentrations of CCL4 (0–30 ng/mL). CM was applied to LECs for 16 h and cell migration was examined by Transwell migration assay. Each experiment was performed three times (N=3). *, $p < 0.05$ as compared with controls. #, $p < 0.05$ as compared with the group treated with CCL4 (30 ng/mL).

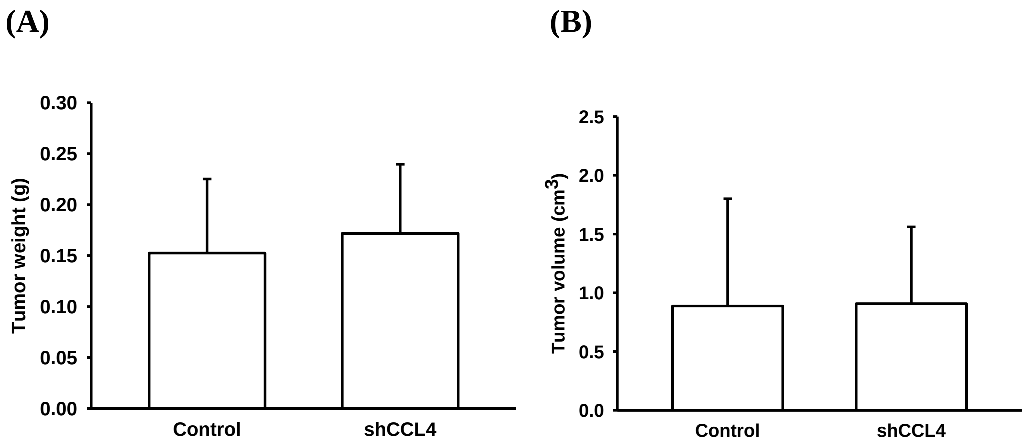


**Figure S3: Effect of CCL4 in murine tumors.** (A-B) SAS cells stably expressing control shRNA (n=12) or CCL4-shRNA (n=12) were established. After 12 days, the mice were sacrificed and the tumors were excised.

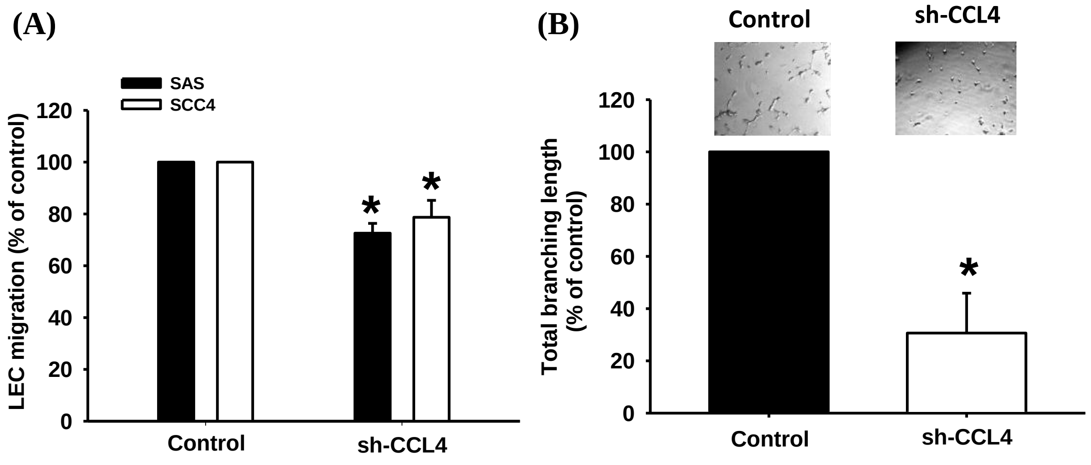


**Figure S4: Inhibiting CCL4 expression suppresses lymphangiogenesis in human OSCC cells.** (A-B) OSCC cells were transfected with CCL4 shRNA; CM was applied to LECs for 24 h. LEC capillary-like structure formation and cell migration were examined by tube formation assay and the Transwell migration assay, respectively. Each experiment was performed three times (N=3). *, $p < 0.05$ as compared with controls.
